# Sarcoidosis presenting as granulomatous myositis in a 16-year-old adolescent

**DOI:** 10.1186/s12969-016-0121-5

**Published:** 2016-11-10

**Authors:** Amir B. Orandi, Eric Eutsler, Cole Ferguson, Andrew J. White, Maleewan Kitcharoensakkul

**Affiliations:** 1 Department of Pediatrics, Division of Rheumatology, Washington University School of Medicine, One Children’s Place, Campus Box 8116, St. Louis, MO USA; 2 Department of Radiology, Division of Pediatric Radiology, Washington University School of Medicine, St. Louis, MO USA; 3 Department of Pathology and Immunology, Washington University School of Medicine, St. Louis, MO USA

**Keywords:** Sarcoidosis, Granuloma, Myositis, Hypercalcemia, Adalimumab

## Abstract

**Background:**

Sarcoidosis is a multi-system disease characterized by the presence of non-caseating epithelioid granulomas in affected tissues, including skeletal muscle. These organized collections of immune cells have important pathophysiologic action including cytokine production leading to inflammation as well as enzymatic conversion of cholecalciferol to calcitriol via 1-α hydroxylase. There are limited reports of isolated granulomatous myositis causing hypercalcemia in pediatric patients. Our patient uniquely presented with symptoms from hypercalcemia and renal insufficiency caused by an overwhelming burden of granulomatous myositis in her lower extremities, but was otherwise asymptomatic.

**Case presentation:**

A 16 year old Caucasian female presented with protracted symptoms of fatigue, nausea and prominent weight loss with laboratory evidence of hypercalcemia and renal insufficiency. She lacked clinical and physical findings of arthritis, weakness, rash, uveitis, fever, lymphadenopathy or respiratory symptoms. After extensive negative investigations, re-examination yielded subtle soft tissue changes in her lower extremities, with striking MRI findings of extensive myositis without correlative weakness or serum enzyme elevation. Biopsy showed the presence of non-caseating epithelioid granulomas and calcium oxalate crystals. The patient responded well to prednisone and methotrexate but relapsed with weaning of steroids. She reachieved remission with addition of adalimumab.

**Conclusions:**

Sarcoidosis should be considered in patients presenting with symptomatic hypercalcemia with no apparent causes and negative routine workup. The absences of decreased muscle strength or elevated muscle enzymes do not preclude the diagnosis of granulomatous myositis.

## Background

Sarcoidosis is a multi-system autoimmune disease characterized by the presence of non-caseating epithelioid granulomas in various organs and tissues. In young children, there is a distinct form of the disease best known as Blau syndrome which presents with a characteristic triad of granulomatous dermatitis, uveitis and polyarticular synovitis and tenosynovitis [1]. Older children with sarcoidosis typically display clinical disease features similar to adults with predominantly pulmonary disease and lymphadenopathy with frequent constitutional symptoms [2]. Despite these clinical phenotypes, there is significant variation in the clinical symptoms of the disease and diagnosis has long relied on histopathology. We herein report on an unusual presentation of sarcoidosis in a 16-year-old adolescent.

## Case presentation

A 16-year-old previously healthy Caucasian female presented with fatigue, nausea and weight loss of 10 kg over a three month period. She had no fever, night sweats, myalgia, arthralgia, rash or pulmonary symptoms. There was no history of sick contacts or recent travel. Apart from visible fatigue and clinically apparent weight loss, her physical examination revealed normal cardiopulmonary exams, and no hepatosplenomegaly, arthritis or skin findings. Initial laboratory studies revealed a white blood count of 7.2 K/cumm, hemoglobin of 12.1 g/dl, hematocrit of 36.4 %, platelets of 315 K/cumm with normal absolute numbers of neutrophils and lymphocytes. Chemistries were notable for total serum calcium of 13.1 mg/dl (reference 8.6-10.3 mg/dl), ionized calcium of 6.16 mg/dl (reference 3.9-5.2 mg/dl), and creatinine of 1.2 mg/dl. Her phosphorus and magnesium were within normal limits. The urine calcium to creatinine ratio was elevated with a ratio of 0.85. These results were followed with additional testing showing an intact parathyroid hormone of 5 pg/mL (reference 14–72 pg/ml), elevated 1–25 Vitamin D level of 168 ng/ml (reference 24–86 pg/ml) and an elevated angiotensin converting enzyme (ACE) level of 268 Units/L (reference 10–55 Units/L). These findings raised concern for granulomatous disease. A broad infectious evaluation for tuberculosis (by PPD and Quantiferon), histoplasmosis, blastomyces, syphilis, and trichinella were negative. Stool calprotectin was negative. A chest radiograph was negative for lymphadenopathy or parenchymal changes. A contrasted CT scan of the chest, abdomen and pelvis also showed no lymphadenopathy or hepatosplenomegaly. A bone marrow biopsy showed hypocellular marrow with erythroid hyperplasia, but absence of malignancy or granulomas. Other serologies showed antinuclear antibody (ANA) positive titer of 1:320 in speckled pattern and negative anti-neutrophil cytoplasmic antibodies (ANCA). She was given intravenous hydration up to 3 L/m^2^ of body surface area to maintain a normal calcium level.

**Fig. 1:**
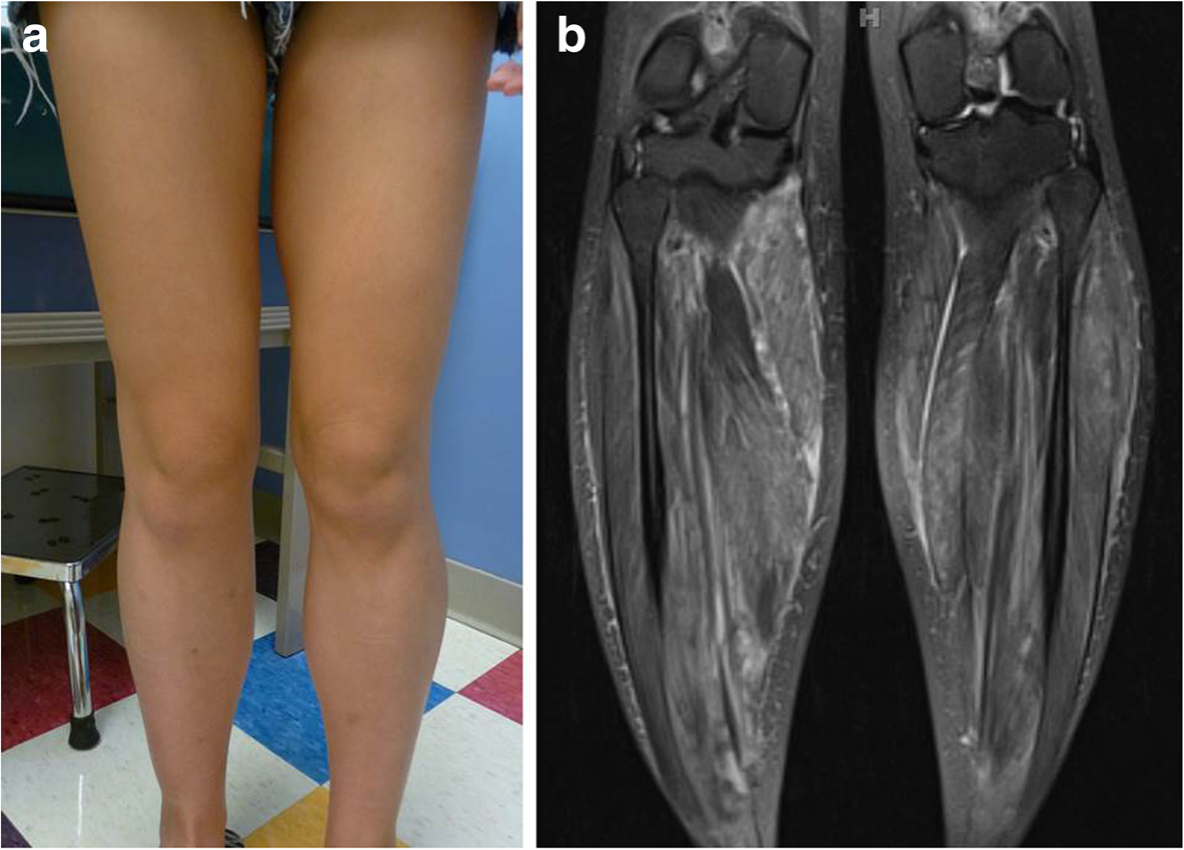
**a** Photograph of lower extremity hypertrophy. **b** Comparison to coronal T2 MRI showing symmetric hypertrophy of lower extremity musculature

**Fig. 2:**
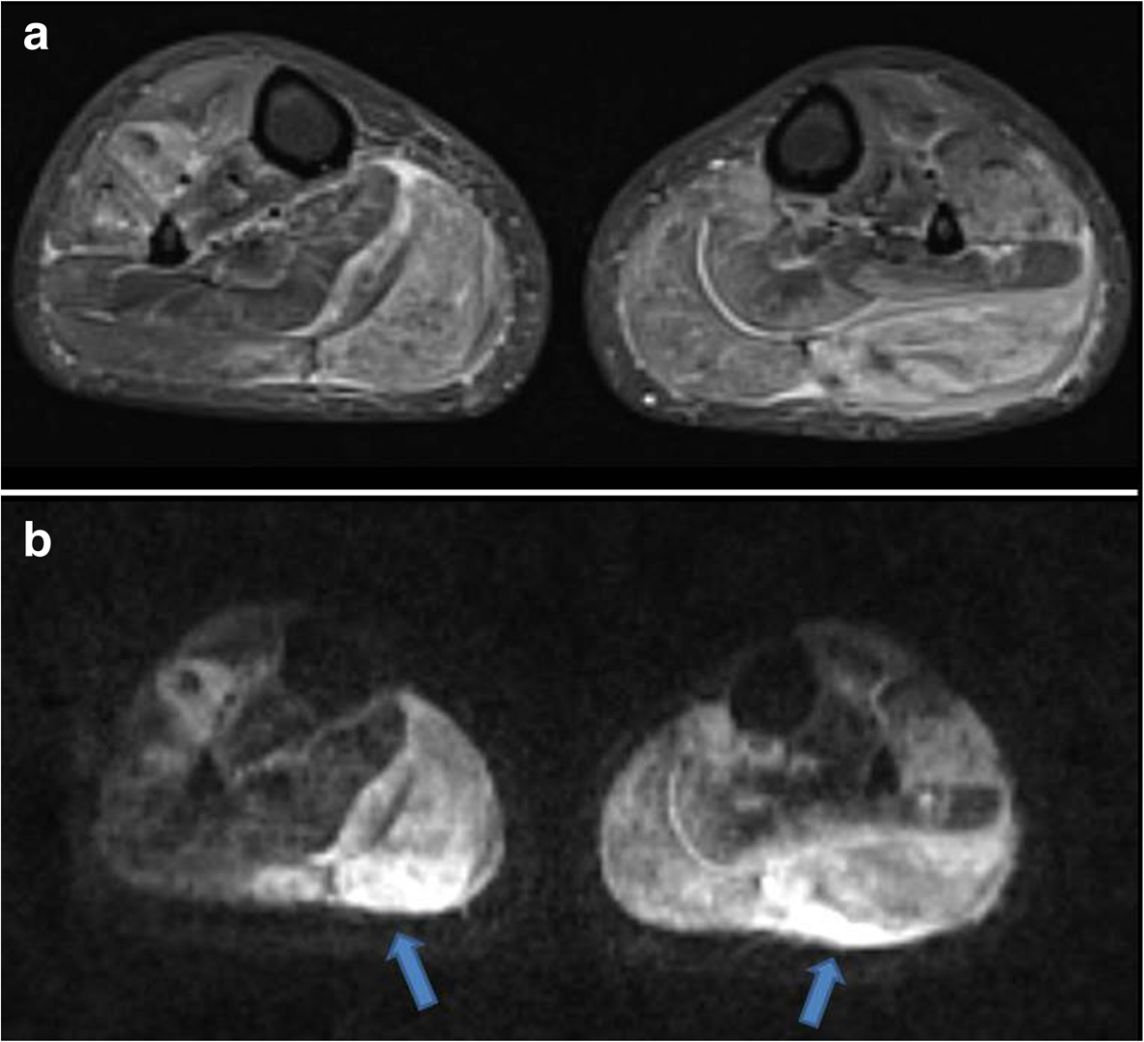
**a** Axial short tau inversion recovery (STIR) image of both legs demonstrates extensive myositis bilaterally with extensive edema involving all of the calf muscles within the anterior, posterior and lateral compartments. **b** Diffusion-weighted sequence reveals patchy areas of restricted diffusion throughout multiple muscles in both legs *(arrows)*, also consistent with acute inflammation. Also note associated intermuscular fascial edema

**Fig. 3:**
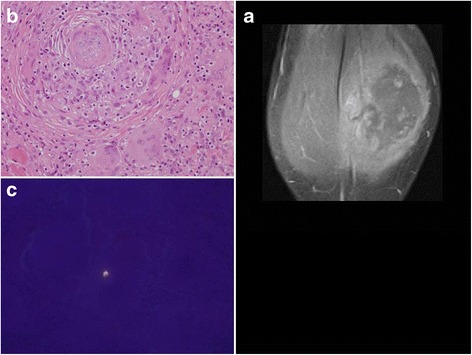
**a** Coronal contrast-enhanced T1 image with fat saturation shows an ill-defined, mass-like area in the lateral head of the left gastrocnemius muscle with central intermediate signal, central non-enhancement, and peripheral hyper-enhancement. Biopsy was taken from area of area. **b** Skeletal muscle involved by severe granulomatous inflammation with osteoclast-like giant cells. **c** Polarization showing crystalline material compatible with calcium oxalate

During her evaluation, the patient continued to be symptomatic from hypercalcemia with fatigue, nausea and vomiting and had progressive normocytic anemia to a nadir of hemoglobin 9.1 g/dl with elevated erythrocyte sedimentation rate (ESR) of 31 mm/h (reference < 20 mm/h). A repeat physical examination revealed soft tissue enlargement with a firm consistency bilaterally in the lower extremities distal to the knees (Fig. 1a). There were no overlying skin changes or tenderness to palpation. Strength and deep tendon reflexes of the extremities were preserved. Plain radiographs and ultrasound showed soft tissue enlargement and muscular hypertrophy respectively without focal changes or calcifications. Laboratory testing for myositis showed creatine kinase of 23 Units/L (reference < 300 Units/L), aldolase 5.7 Units/L (reference < 14.7 Units/L), lactate dehydrogenase (LDH) of 177 Units/L (reference 100–200 Units/L) and normal serum transaminases. Magnetic resonance imaging (MRI) of the bilateral lower extremities revealed extensive myositis involving every muscle in both legs (Figs. 1b and 2). There was a mass-like area in the lateral head of the left gastrocnemius muscle (Fig. 3a). Needle electromyography (EMG) showed fibrillations and positive sharp waves in the right medial gastrocnemius and myopathic polyphasic potentials with mild early recruitment compatible with myopathy. Nerve conduction studies were normal. A biopsy was obtained with gross tissue intraoperatively described as pale and edematous. Microscopy showed a destructive macrophage-rich process with well-formed non-necrotizing granulomas, numerous giant cells and sparse residual muscle. Some giant cells contained polarizable crystalline material most compatible with calcium oxalate (Fig. 3b and c). Special studies were employed to exclude Langerhans cell histiocytosis and infection with fungal or acid fast organisms. A slip lamp and dilated eye exam did not demonstrate uveitis. Spirometry including lung volume and diffuse lung capacity were normal. Echocardiogram showed no abnormalities. Based on her clinical symptoms, pathology findings, and the exclusion of other causes, the diagnosis of sarcoidosis was made. She was initiated on a prednisone taper and weekly subcutaneous methotrexate at 15 mg/m^2^, with improvement in her symptoms of fatigue and nausea and normalization of her calcium and ACE levels. However, due to difficulty weaning off steroids, adalimumab 40 mg every 2 weeks was added three months after diagnosis with sustained improvement.

## Conclusion

Pediatric sarcoidosis is rare with an estimated incidence of 0.2 per 100, 000 per year. The mean age of onset is 13–15 years, but presentations may vary with age [1, 2]. The hallmark finding of sarcoidosis are the presence of non-caseating epithelioid granulomas, which are believed to be an antigen-driven cell-mediated immune response involving CD4+ T cells that eventually differentiate into Type 1 T helper (Th1) cells which then secrete IL-2 and IFN-γ, supporting macrophage TNF-α production, promoting the cellular immune response [3, 4]. The recruited and organized immune cell collections include activated macrophages that have been shown to produce 1-α hydroxylase which converts cholecalciferol (25-OH Vitamin D) to calcitriol (1, 25-dihydroxy Vitamin D) [5]. Calcitriol then increases intestinal calcium absorption and bone resorption leading to hypercalcemia and/or hypercalciuria, known to be frequent features of the disease particularly in those with high granuloma burden. Epithelioid cells of the granulomata produce angiotensin converting enzyme (ACE), a controversial biomarker of sarcoidosis that is often elevated, but lacks diagnostic specificity and sensitivity [6, 7]. Thus, some laboratory findings could be suggestive of granuloma presence and activity, but pursuit must be taken to find tissue for histopathologic confirmation and diagnosis. In a recent review of 101 adult cases of calcitriol-mediated hypercalcemia, approximately half were eventually diagnosed with sarcoidosis [8]. Overall, granulomata are an infrequent finding on skeletal muscle biopsies [9]. When isolated, sarcoidosis and idiopathic granulomatous myositis are the two most frequent causes, respectively. Symptomatic presentations of sarcoid myopathy have multiple phenotypes including nodular and atrophic forms and are more often from chronic disease [10]. The frequency of asymptomatic granulomatous myositis in sarcoidosis has been estimated between 50 and 80 % and occurs more often in early stages of the disease [11]. Our case underscores the importance of considering sarcoidosis or granulomatous myositis in patients who present with hypercalcemia of unclear etiology and reinforces the need for tissue diagnosis and exclusion of other causes. MRI showed excellent utility in detecting granulomatous myositis in patients without typical myositis features and anti-TNF monoclonal antibody therapy was efficacious in our refractory case.

## Abbreviations

ACE: Angiotensin-converting enzyme
ANA: Antinuclear antibody
ANCA: Anti-neutrophil cytoplasmic antibodies
EMG: Electromyography
ESR: Erythrocyte sedimentation rate
LDH: Lactate dehydrogenase
MRI: Magnetic-resonance imaging
